# Alcohol-Induced Blood-Brain Barrier Impairment: An In Vitro Study

**DOI:** 10.3390/ijerph18052683

**Published:** 2021-03-07

**Authors:** Donatello Carrino, Jacopo Junio Valerio Branca, Matteo Becatti, Ferdinando Paternostro, Gabriele Morucci, Massimo Gulisano, Lorenzo Di Cesare Mannelli, Alessandra Pacini

**Affiliations:** 1 Department Experimental and Clinical Medicine, Anatomy and Histology Section, University of Firenze, 50134 Firenze, Italy; donatello.carrino@unifi.it (D.C.); jacopojuniovalerio.branca@unifi.it (J.J.V.B.); ferdinando.paternostro@unifi.it (F.P.); massimo.gulisano@unifi.it (M.G.); 2 Department of Experimental and Clinical Biomedical Sciences “Mario Serio”, University of Firenze, 50134 Firenze, Italy; matteo.becatti@unifi.it; 3 Department of Translational Research and New Technologies in Medicine and Surgery, University of Pisa, 56126 Pisa, Italy; gabriele.morucci@unipi.it; 4 Department of Neuroscience, Psychology, Drug Research and Child Health (NEUROFARBA), Pharmacology and Toxicology Section, University of Firenze, 50139 Firenze, Italy; lorenzo.mannelli@unifi.it

**Keywords:** alcoholism, alcohol abuse, oxidative stress, blood–brain barrier, tight junction

## Abstract

In recent years, alcohol abuse has dramatically grown with deleterious consequence for people’s health and, in turn, for health care costs. It has been demonstrated, in humans and animals, that alcohol intoxication induces neuroinflammation and neurodegeneration thus leading to brain impairments. Furthermore, it has been shown that alcohol consumption is able to impair the blood–brain barrier (BBB), but the molecular mechanisms underlining this detrimental effect have not been fully elucidated. For this reason, in this study we investigated the effects of alcohol exposure on a rat brain endothelial (RBE4) cell line, as an in vitro-validated model of brain microvascular endothelial cells. To assess whether alcohol caused a concentration-related response, the cells were treated at different times with increasing concentrations (10–1713 mM) of ethyl alcohol (EtOH). Microscopic and molecular techniques, such as cell viability assay, immunofluorescence and Western blotting, were used to examine the mechanisms involved in alcohol-induced brain endothelial cell alterations including tight junction distribution, apoptosis, and reactive oxygen species production. Our findings clearly demonstrate that alcohol causes the formation of gaps between cells by tight junction disassembly, triggered by the endoplasmic reticulum and oxidative stress, highlighted by GRP78 chaperone upregulation and increase in reactive oxygen species production, respectively. The results from this study shed light on the mechanisms underlying alcohol-induced blood–brain barrier dysfunction and a better understanding of these processes will allow us to take advantage of developing new therapeutic strategies in order to prevent the deleterious effects of alcohol.

## 1. Introduction

In the last couple of years, alcohol has been listed as one of the main dependent drugs worldwide. Data from the World Health Organization (WHO) indicate that more than 3 million people died as a result of harmful use of alcohol in 2016 [1], including it as one of the leading risk factors for population health worldwide.

Alcohol abuse not only impairs liver function (steatosis, hepatitis and cirrhosis) [2], but also can lead to cardiovascular diseases [3], malignant neoplasms [4], infectious diseases [5] and digestive disorders (such as pancreatitis) [6,7]. Moreover, since alcohol has been listed as a psychoactive substance, it induces mental disorders such as depression and psychoses. Indeed, alcohol intoxication has been observed to induce neuroinflammation and neurodegeneration in humans and animals [8,9,10]. Recently, many neurological disorders have been associated to alcohol abuse such as Parkinson’s disease, epilepsy, and many others [11,12,13,14].

Neurodegenerative disorders are debilitating pathologies affecting both the central and peripheral nervous systems and represent a growing health issue worldwide [15]. However, the etiopathogenesis of such debilitating diseases needs to be clarified and deeply investigated. In the last decade, many hypotheses have come to light including the inflammatory hypotheses involving the glial compartment [16,17,18] and the blood–brain barrier (BBB) alterations [19,20,21].

The BBB plays a pivotal role in discerning harmful from harmless substances in the bloodstream entering the brain parenchyma [22]. It is characterized by the presence of different cellular components such as endothelial cells, pericytes and astrocyte end-feet that are all essential for the correct BBB function [23,24,25]. Notably, endothelial cells are sealed to each other by the presence of tight junctions’ proteins, avoiding the entrance of toxic molecules that can alter the brain physiology [26,27]. Indeed, many studies have underlined the primary role of endothelial cells in maintaining the homeostasis of the central nervous system, whose impairment leads to neurodegeneration [19,21,28,29,30].

Previous studies have clearly indicated that chronic and excessive ethanol (EtOH) consumption may enhance oxidative injury of neural cells by increasing reactive oxygen species (ROS) production [31,32]. In addition, oxidative stress was demonstrated to increase BBB permeability after different toxic stimuli both in vitro and in vivo models [33,34]. Overall, little is known about the mechanisms involved in BBB alterations during excessive alcohol consumption.

Using a rat brain endothelial (RBE4) cell line, and our previously developed in vitro BBB model [35,36], we demonstrated that EtOH decreased Zonula Occludens 1 (ZO-1) tight junction (TJ) integrity via induction of endoplasmic reticulum (ER) stress and Bcl-2-associated X protein (BAX) expression. Additionally, the current study addresses the role of oxidative stress in ZO-1 alteration caused by Et-OH exposure. Finally, our data demonstrate a clear link amongst RBE4 alcohol metabolism, oxidative stress, and ZO-1 and cytoskeletal protein alterations.

## 2. Materials and Methods

### 2.1. Cell Culture and Treatments

Rat brain endothelial cell line (RBE4) cultures were maintained in 75 cm^2^ flasks (Euroclone, Milan, Italy) as previously described [35,36]. In brief, cells were cultured in alpha-MEM/Ham’s F10 (1:1 ratio) supplemented with 1 ng/mL (bFGF) (GIBCO, Thermo Fisher Scientific, Milan, Italy), 10% FBS, 1% penicillin/streptomycin (Euroclone, Milan, Italy) at 37 °C, 5% CO_2_ in humidified atmosphere.

To assess whether alcohol caused a concentration-related response, cells were treated with increasing concentrations (10, 35, 50, 75, 100, 171.3 or 1713 mM) of ethyl alcohol (EtOH) at different times. These concentrations are equal or equivalent to two or three times the legal limits for blood alcohol concentration in Italy (corresponding to 0.5 g/L as declared on the D. L. 30 aprile 1992, n. 285, art. 186) (http://www.mit.gov.it/mit/site.php?p=normativa&o=vd&id=1&id_cat=&id_dett=0 (accessed on 7 March 2021)).

The EtOH withdrawal experiments were performed replacing the medium with EtOH-free medium for the following 24 and 48 h.

### 2.2. MTT Assay

RBE4 cells were gently seeded at a density of 2.5 × 10^4^ cells/well in 96-well plates in growth medium for 24 h. The following day, the medium was replaced with starvation medium (without FBS and bFGF) and RBE4 cells were treated with different concentrations of EtOH for 30, 60, 120, 240 min. Cell viability was evaluated using the MTT (3-(4,5-di-methylthiozol-2-yl)-2,5-diphenyltetrazolium bromide) assay, based on the reduction of the tetrazolium salt to formazan by mitochondrial dehydrogenases. After the treatments, 1 mg/mL of MTT solution (Sigma Aldrich, Milan, Italy) was added into each well at the final volume of 100 µL. After 30 min, formazan crystals were dissolved by adding 100 μL of dimethyl sulfoxide (DMSO) (Sigma Aldrich, Milan, Italy) and optical density was measured at 570 nm using a microplate reader (MultiskanFC™ microplate photometer, ThermoFisher Scientific, Milan, Italy).

### 2.3. Western Blotting

Cells were gently seeded in 100 mm ∅ Petri dishes (Euroclone, Milan, Italy) with the appropriate growth medium for 24 h, at a density of 4 × 10^6^ cells/dish. The following day, the culture medium was substituted with starvation medium and different concentrations of alcohol were added. After stimulation, cells were harvested and lysed by incubation for 30 min at 4 °C in RIPA buffer (Euroclone, Milan, Italy) and protease inhibitor cocktail (Sigma Aldrich, Milan, Italy) according to the manufacturer’s instructions. Cellular debris were removed by centrifugation at 12,000 revolutions per minute (rpm) for 10 min at 4 °C. After determination of protein concentration in each sample by bicinchoninic acid (BCA) protein assay (Sigma Aldrich, Milan, Italy), 30 µg of the total proteins was loaded on 4–12% acrylamide/bis-acrylamide (Euroclone, Milan, Italy) gels, and then transferred to nitrocellulose membranes (Porablot NPC, MACHEREY-NAGEL, Milan, Italy). The blotted proteins were blocked with bovine serum albumin (BSA; CliniSciences, Guidonia Montecelio, Italy) at 3% in tween-tris buffered saline (tTBS) solution for 30 min at room temperature (RT). After that, the nitrocellulose membranes were soaked with proper primary antibody as reported in Table 1 diluted in blocking solution, overnight at 4 °C. The following day, appropriate HRP-conjugated secondary antibodies (Santa Cruz Biotechnology, Santa Cruz, CA, USA) were used at 1:5000 dilution for 1 h at RT. Protein bands were detected by ECL Plus Western Blotting Detection Reagent (GE Healthcare, Milan, Italy). Band density was determined using ImageJ software v. 1.52a (National Institute of Health, Bethesda, USA). β-actin or α-tubulin were used as internal loading control to normalize the expression of proteins of interest.

### 2.4. Measurement of ROS 

RBE4 cells were gently seeded into a 96-well plate and allowed to adhere for 24 h, at 2.5 × 10^4^ cells/well. The following day, after removing growth medium, cells were loaded with 5 μM CM-H_2_DCFDA (Life Technologies, ThermoFisher Scientific, Milan, Italy) for 30 min at 37 °C in 5% CO_2_, protected from light. After washing with PBS, the cells were exposed to treatments: control (PBS only; 0 mM EtOH), 50 mM EtOH, 75 mM EtOH or 100 mM EtOH. The fluorescence, resulting from the conversion of dichlorofluorescin diacetate (DCFH-DA) to dichlorofluorescein (DCF), was measured after 30, 120, 240 min using a VICTOR microplate reader (PerkinElmer, Milan, Italy) set to an excitation wavelength of 485 nm and an emission wavelength of 530 nm. The amount of fluorescence was directly correlated to the amount of ROS within the cells.

### 2.5. Immunofluorescent Labelling

RBE4 cells were gently seeded at a density of 5 × 10^4^ on sterilized coverslips, lodged in 6-well plates, in complete growth medium for at least 24 h. After growing to the required density, RBE4 were stimulated and then fixed with 4% formaldehyde for 10 min at RT or with cold methanol for 20 min. Cells were permeabilized with 0.1% Triton X-100 (Sigma Aldrich, Milan, Italy) in PBS for 10 min, blocked with 1% BSA in 0.1% Triton X-100 in PBS for 30 min and incubated with ZO-1 primary antibody (ThermoFisher Scientific, Milan, Italy) diluted in blocking solution (1:50), overnight at 4 °C. The following day, the cover slips were washed three times in PBS and incubated with the appropriate AlexaFluor 488 secondary antibodies (Invitrogen, ThermoFisher Scientific, Milan, Italy) at 1:200 dilution in blocking solution for 1 h at RT. Cells were counterstained with DAPI (4′,6-diamidin-2-fenilindolo; 1:2000 dilution; Invitrogen, Milan, Italy) to visualize cellular nuclei. Samples were examined under a fluorescence microscope (Leica DM6000B microscope equipped with a DFC350FX camera) at the total magnification of 400×.

### 2.6. Statistical Analysis

Each experimental data was expressed as mean ± S.E.M. (standard error of the mean).

For MTT assay, we performed experiments three times in quintuplicate.

For Western blotting analysis and ROS evaluation, each experiment was performed three times, in triplicate.

For immunofluorescence staining analysis, we performed three different experiments and fifteen images per experimental point were captured.

Statistical analysis was performed with one-way ANOVA followed by Dunnett post hoc test. 

In each comparison, differences were considered significant when *p* value was less than 0.05 (* *p* < 0.05).

## 3. Results

### 3.1. RBE4 Cell Viability

**Figure 1:**
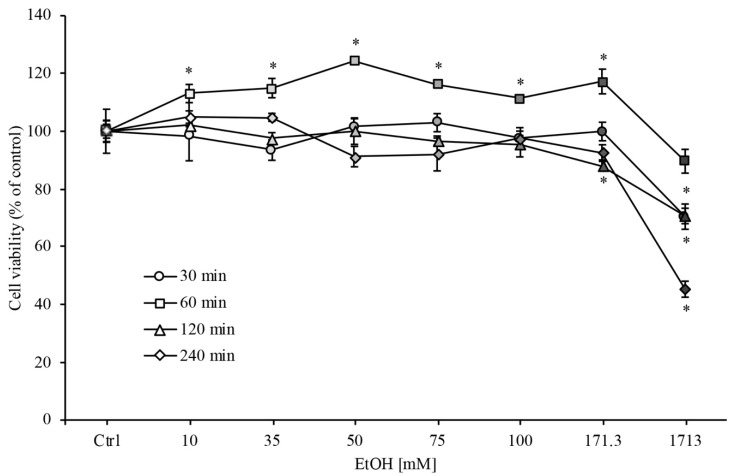
RBE4 cell viability assay during ethanol (EtOH) treatment. RBE4 cells were incubated with increasing EtOH concentrations (10–1731 mM) for 30 min, 1, 2 and 4 h. Cell viability, quantified by MTT (3-(4,5-di-methylthiozol-2-yl)-2,5-diphenyltetrazolium bromide) assay (570 nm wavelength absorbance), decreased significantly (* *p* < 0.05) only at the highest EtOH concentrations. Values are expressed in percentage of control (untreated cells) absorbance as the mean ± S.E.M. of three independent experiments in quintuplicate. Grayscale refers to the increasing EtOH concentration. * *p* < 0.05 vs. control (untreated cells).

In order to evaluate the EtOH effects on RBE4 cells viability, we performed the MTT assay with different concentrations of EtOH (ranging from 10 to 1713 mM) at different timepoints (from 30 min to 2 h). As clearly shown in Figure 1, RBE4 cell viability decreased significantly (* *p* < 0.05) only at the highest EtOH concentration (1713 mM). A slight but significant increase in cell viability at 1 h timepoint was evidenced that could be attributed to an increase in cellular metabolism because alcohol is an energy-yielding nutrient.

On the other hand, for EtOH concentrations ranging from 35 to 100 mM, cell viability was unchanged in respect to control levels. In addition, the EtOH concentrations and the time of exposure used in this study are similar to those seen in the peripheral blood of moderately to severely intoxicated humans [37,38], and in vivo experimental models of chronic alcohol abuse [39,40]. For these reasons, these concentrations were used in all subsequent experiments.

### 3.2. Evaluation of ROS Generation

**Figure 2:**
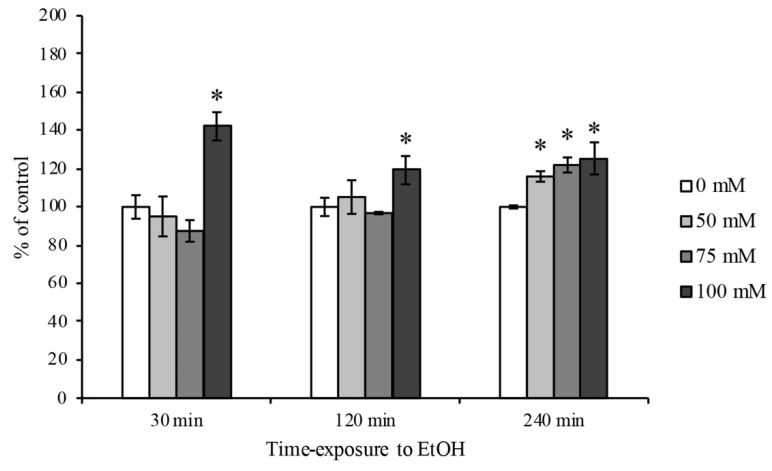
ROS quantification on RBE4 cells after EtOH treatment. Representative ROS production by H_2_DCFDA fluorescence probe after EtOH (50, 75 and 100 mM) treatment at 30 min, 2 and 4 h timepoints. The reported values are expressed in percentage of control (untreated cells) as the mean ± S.E.M. of three independent experiments in triplicate. * *p* < 0.05 vs. control for each timepoint.

The intracellular ROS quantification in RBE4 cells was carried out by a chemiluminescent analysis. As reported in Figure 2, the EtOH intoxication induced a first ROS production peak at the highest concentration (100 mM) at 30 min timepoint in comparison to control levels. Furthermore, a significant increase in ROS production after 4 h timepoint has been observed at all EtOH concentrations used. 

### 3.3. BAX Protein Expression Levels

**Figure 3:**
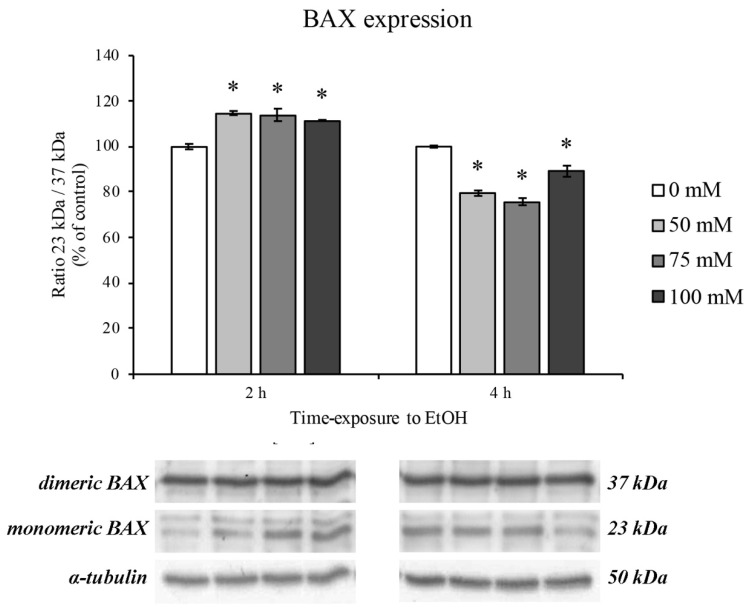
Bcl-2-associated protein (BAX) expression on RBE4 cells after EtOH treatment. Representative Western blotting analysis of the EtOH (50–100 mM) effects on the protein levels of dimeric (upper bars) and monomeric (lower bars) BAX after 2 and 4 h of treatment. Value bars are expressed in percentage of control (untreated cells) as the mean ± S.E.M. of three independent experiments in triplicate. * *p* < 0.05 vs. control.

The protein expression of BAX was used in order to evaluate cell apoptosis after 2 and 4 h treatment with increasing concentrations of EtOH. In this regard, we focused on the balance between BAX dimers (37 kDa) and monomers (23 kDa). As reported by Garner and colleagues, when BAX is present in dimeric form, BAX-mediated apoptosis induction is hindered, whereas the presence of monomeric BAX triggers the apoptotic cascade [41]. As shown in Figure 3, the ratio between the monomeric and dimeric form significantly increased after 2 h of treatment. On the contrary, after 4 h of EtOH treatment, BAX balance moved towards the dimeric form, showing a decrease in monomeric and dimeric ratio, suggesting for an autoinhibited apoptosis. 

**Figure 4:**
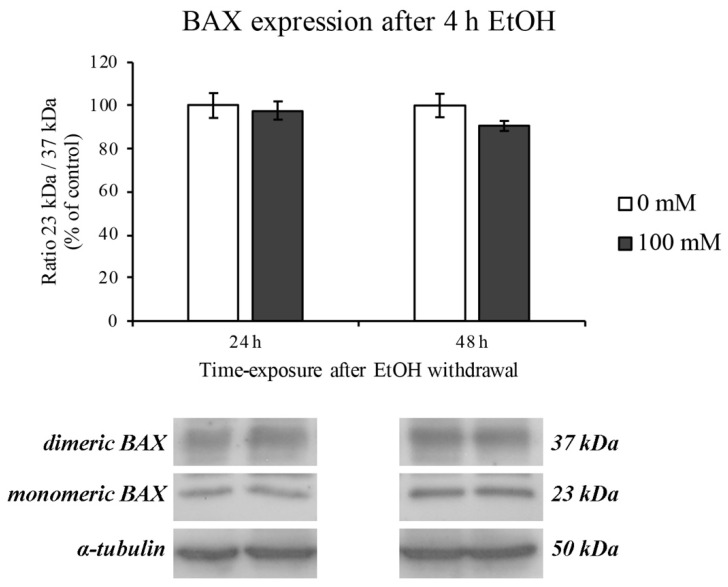
BAX expression on RBE4 cells after EtOH withdrawal. Representative Western blotting analysis after EtOH 100 mM withdrawal for 24 and 48 h. Value bars are expressed in percentage of control (untreated cells) as the mean ± S.E.M. of three independent experiments in triplicate.

These events were completely restored after EtOH withdrawal for the following 24 and 48 h (Figure 4). 

### 3.4. EtOH-Dependent ER Stress

Since a pivotal role in oxidative stress has been attributed both to mitochondrial and ER stress [42], we evaluated the GRP78 protein expression as marker of ER dysfunction.

**Figure 5:**
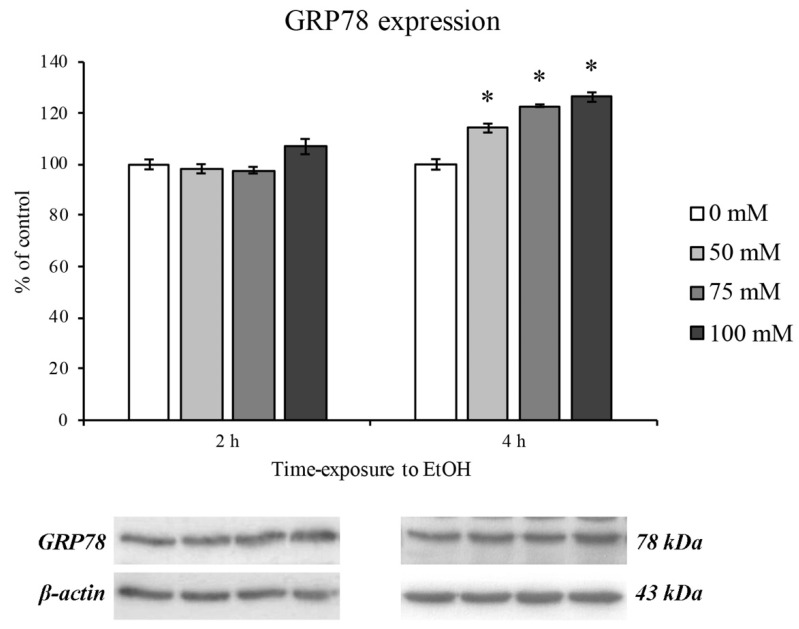
ER stress (GRP78 expression) on RBE4 cells after EtOH treatment. Western blotting analysis and quantification of GRP78 expression during EtOH (50, 75 and 100 mM) treatments at 2 and 4 h timepoints. Values are expressed in percentage of control (untreated cells) as the mean ± S.E.M. of three independent experiments in triplicate. * *p* < 0.05 vs. control.

After 2 h of EtOH treatment no GRP78 increase was observed, supporting the hypothesis that alcohol is utilized by the cell as a metabolite [43]. On the contrary, an ER stress response was evidenced after 4 h of treatment. Moreover, the GRP78 expression levels increased as with increasing concentrations of EtOH in a dose-dependent manner (Figure 5). 

**Figure 6:**
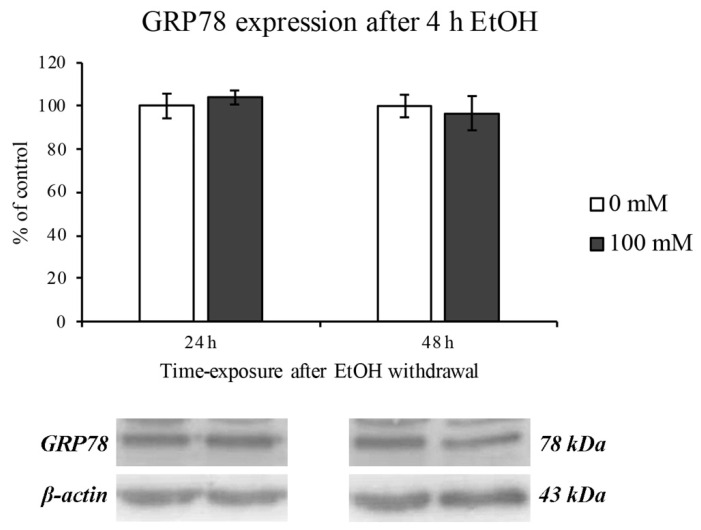
ER stress (GRP78 expression) on RBE4 cells after EtOH withdrawal. Western blotting analysis and quantification of GRP78 expression levels after 24 and 48 of EtOH 100 mM (4 h treatment) withdrawal. Values are expressed in percentage of control (untreated cells) as the mean ± S.E.M. of three independent experiments in triplicate.

The EtOH-induced ER stress was completely restored after 24 and 48 h of EtOH withdrawal, as reported in Figure 6.

### 3.5. Expression of Antioxidant Enzymes

**Figure 7:**
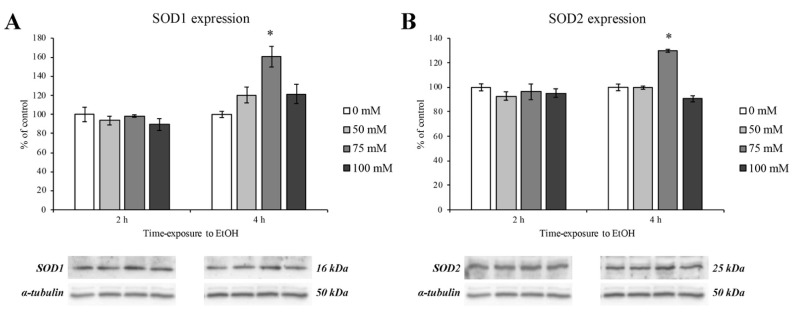
SOD1 and SOD2 expression levels on RBE4 cells after EtOH treatment. Western blotting analysis and quantification of SOD1 (panel **A**) and SOD2 (panel **B**) protein expression levels during EtOH (50, 75 and 100 mM) treatments at 2 and 4 h. Values are expressed in percentage of control (untreated cells) as the mean ± S.E.M. of three independent experiments in triplicate. * *p* < 0.05 vs. control.

In order to evaluate the oxidative stress response after EtOH treatment, we evaluated the levels of superoxide dismutase proteins (SOD1 and SOD2) at 2 and 4 h timepoints (Figure 7, panels A and B, left and right panel, respectively). The results obtained by Western blotting revealed a significant upregulation of SOD1 and SOD2 protein expression and only at the concentration of 75 mM and after 4 h of EtOH exposure (Figure 7, panels A and B, dark grey columns).

As previously demonstrated, SOD’s activity is abolished at the highest EtOH concentration and time exposure, thus addressing the deleterious effect of prolonged alcohol abuse [44].

### 3.6. The Effect of EtOH on the Tight Junction Protein ZO-1

It has been known that alcohol can reach the brain parenchyma inducing cognitive alteration and impairment depending on its blood stream concentration. Since the brain is protected and enveloped by microvascular endothelial cells that are tightly sealed by tight junction proteins, we performed our last experiments on the evaluation of ZO-1 tight junction protein expression and staining pattern. Actually, ZO-1 protein is needed as an anchor point for the claudin and occludin proteins [45] and is necessary for TJ formation [46]. Because of the specificity of this transmembrane protein to the BBB, it is often used as markers for successful BBB formation.

**Figure 8:**
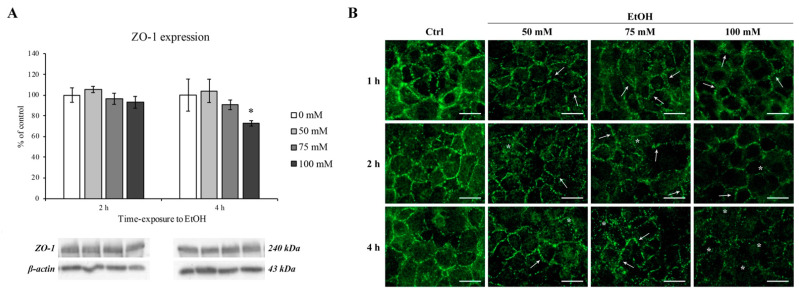
ZO-1 expression and immunofluorescent staining on RBE4 cells after EtOH treatment. (**A**) Western blotting analysis and quantification of ZO-1 after EtOH (50, 75 and 100 mM) exposure at 2 and 4 h. Values are expressed in percentage of control (untreated cells) as the mean ± S.E.M. of three independent experiments in triplicate. * *p* < 0.05 vs. control. (**B**) Changes in ZO-1 distribution were evaluated after EtOH (50, 75 and 100 mM) treatments at 1, 2, and 4 h in comparison to control (untreated cells). Arrows show the “zip-like” structure as indicative for morphological alterations in intercellular junctions. Asterisks show the presence of holes formed between endothelial cells. Scale bar: 25 µm.

As shown in Figure 8, ZO-1 expression was significantly decreased only at the highest EtOH concentration (100 mM) and at 4 h timepoint (panel A, right side). 

On the other hand, as clearly shown in Figure 8 (panel B), ZO-1 cellular distribution was severely altered after 1 h even at lower concentrations (50 and 75 mM) in comparison with control group where a ZO-1 continuous distribution at the cell–cell borders was observed. 

This scenario deeply changed after EtOH treatment where a gradual transition from “dot-like” to “zipper-like” structure (arrows) as well as an apparent disruption on the normal junctional integrity (asterisks) was evidenced. Moreover, after 4 h of EtOH, the ZO-1 staining pattern was almost disappeared and it is no longer possible to distinguish the cell boundaries, confirmed by the ZO-1 protein downregulation by Western blotting analysis. 

**Figure 9:**
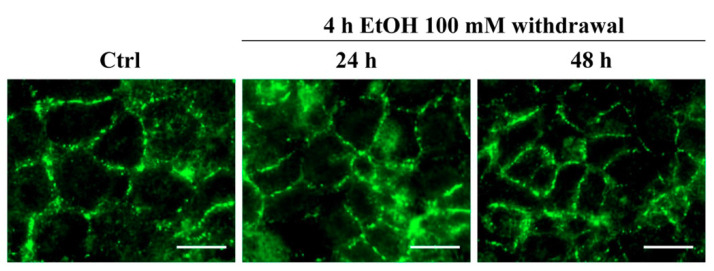
ZO-1 immunofluorescent staining on RBE4 cells after EtOH withdrawal. The ZO-1 staining pattern of distribution evaluated after EtOH withdrawal showing a normal pattern of distribution. Scale bar: 25 µm.

However, if EtOH was removed and replaced with EtOH-free medium for the following 24 and 48 h, the ZO-1 restored its normal pattern of distribution, as shown in Figure 9.

## 4. Discussion

Among the alcohol-associated disorders, those affecting the nervous system can lead to fatal consequence such as traffic injuries [47], suicide [48,49], and interpersonal violence [50]. Furthermore, recent studies have associated alcohol abuse with many neurodegenerative disorders such as Parkinson’s and Alzheimer’s diseases, amyotrophic lateral sclerosis, and epilepsy [11,12,51]. This latter scenario might be explained as an indirect alcohol abuse association and not as a direct consequence. Indeed, once it has entered the bloodstream, alcohol reaches the systemic organs including the brain. 

The brain parenchyma is protected by a tightly sealed and selective wall called BBB. The BBB has the key role to sort out harmless molecules from toxic substances avoiding potential side effects occurring when toxicants enter the nervous system.

In this regard, the role of EtOH-induced permeability of the BBB [51], that in turn induces neurodegenerative disorders and neuroinflammation [19], has been demonstrated by in vivo and post-mortem analysis [52,53]. However, little is known about the signaling pathway triggered by EtOH that induces permeabilization of the BBB. Aiming to better understand the EtOH-dependent molecular pathway, we treated a rat brain endothelial cell line (RBE4) with EtOH and assessed monolayer tightness by expression and modification of ZO-1 TJ protein pattern of distribution as marker of BBB permeability alteration. 

Firstly, we focused on EtOH concentrations ranging from 10 to 1713 mM in order to evaluate cell viability of RBE4 cells. In accordance with our results, and based on previously reported data on different cell lines [39,54,55], we focused on 50, 75 and 100 mM EtOH concentration that also mimics the blood alcohol concentrations observed in social drinkers or chronic alcoholics [56], and it may be hypothesized that EtOH effects were reversible after its withdrawal.

According to previously reported data in different BBB in vitro models [39,54,55,57], our results showed that EtOH rapidly increased ROS formation 30 min after treatment at the highest concentrations and levels remained high until 4 h in comparison to control levels. 

It is well known that the most abundant ROS production occurs when mitochondrial electron transport function is compromised, underlining the mitochondria dysfunction [58,59]. However, since it has been reported that BAX protein expression induces a pro-oxidant state that is upstream of the ROS overproduction [60], our results are in accordance with these data showing BAX overexpression at 2 h of EtOH treatment. Interestingly, this scenario completely changed after 4 h of EtOH where an increase in the BAX dimeric form is highlighted. This latter form of BAX has been recently found to be enrolled for apoptotic molecular pathway autoinhibition [41]. These data lead us to hypothesize that RBE4 cells try to engage a survival mechanism in order to counteract mitochondria dysfunction and ROS overproduction. However, these adverse effects were completely restored after EtOH withdrawal.

As previously reported [61,62], since mitochondria are closely associated to ER, the activation of BAX can elicit the ER stress signaling cascade. Indeed, the measurement of the ER chaperone GRP78 expression levels, a well-known hallmark and a central regulator of ER stress [63,64], showed a modest, but significant increase only after 4 h of treatment. Moreover, the GRP78 upregulation was totally reversed by EtOH withdrawal. Considering that the increment in ER stress is likely due to the increase in the toxic metabolites of alcohol such as acetaldehyde and ROS and that the ROS overproduction could be evoked by ER-mitochondria cross talk [65], our results showing an increase in ROS that coincides with that of GRP78 expression would support this hypothesis.

Furthermore, it is worth noticing that at the last timepoint of this study, the RBE4 cells tried again to counteract the oxidative stress, by increasing the level of antioxidant molecules such as SOD1 and SOD2. It has been reported that the SOD family proteins are produced to counteract oxidative stress scavenging ROS [65]. 

Unfortunately, all these defense mechanisms that endothelial cells put in place to counteract the effect of EtOH were not sufficient to prevent the TJ protein damage. Even if the ZO-1 expression level decreased only after 4 h of 100 mM EtOH, alterations of its subcellular distribution occurred at lower timepoints. Indeed, after 1 h of EtOH exposure, the ZO-1 arrangement, evidenced by immunofluorescence staining, shift from a “dot-” to “zip-like” pattern. The alteration of the ZO-1 distribution pattern is even more marked with increasing EtOH concentrations and exposure times as well as an EtOH-dependent ZO-1 expression downregulation. These alterations were abolished when EtOH was removed from the culture medium.

These deleterious effects could be linked to EtOH action on different cell compartments. Indeed, as previously reported, oxidative stress, mitochondrial dysfunction and ER stress cause TJ dysregulation that led us to hypothesize the increasing permeability in brain endothelial cells [36].

## 5. Conclusions

The results from this study shed light on the mechanisms underlying alcohol-induced brain endothelial cells dysfunction.

Alcohol metabolism in RBE4 cells produces oxidative and ER stress by ROS production and GRP78 chaperone upregulation, respectively, that may lead to TJ disassembly.

A better understanding of these processes could reveal new potential targets for therapy in brain injuries caused by alcohol abuse and in several EtOH-dependent CNS diseases. 

## Figures and Tables

**Table 1:** Primary antibody for Western blotting analysis.

	Dilution	Manifacturer	Host
GRP78	1:500	ThermoFisher Sientific, Milan, Italy	rabbit
ZO-1	1:500	ThermoFisher Sientific, Milan, Italy	rabbit
SOD1 SOD2	1:5000	GeneTex, Prodotti Gianni, Milan, Italy	rabbit
BAX	1:200	Santa Cruz Biotechnology, Santa Cruz, CA, USA	rabbit
β-actin	1:10,000	Santa Cruz Biotechnology, Santa Cruz, CA, USA	mouse
α-tubulin	1:10,000	Santa Cruz Biotechnology, Santa Cruz, CA, USA	mouse
